# Cohort Profile: Our Future Health

**DOI:** 10.1093/ije/dyaf171

**Published:** 2025-10-15

**Authors:** Michael B Cook, Naomi Adams, Ashley Adjetey, Ryan Arathimos, Marko Balabanovic, Ros Blackwood, Adam Booth, Benjamin J Cairns, Ali Connell, Sidra Ellis, Ben Elsworth, Kate Evans, Alice Forman, Eva Gradovich, Cosima Gretton, Fiona Grimm, David J Hunter, Kamil Lipinski, Jodie Lord, Jane Luff, Fiona Maleady-Crowe, Rachel Moran, Sophie North, Alicia Peel, Diana van der Plaat, Kirstin Purves, Fiona Reddington, Andrew Roddam, Saskia C Sanderson, Tim Sprosen, Adam Steventon, Iain Turnbull, Emma Vestesson, Raghib Ali

**Affiliations:** Our Future Health, Manchester, United Kingdom; Imperial College London, London, United Kingdom; Nuffield Department of Population Health, University of Oxford, Oxford, United Kingdom; Our Future Health, Manchester, United Kingdom; Our Future Health, Manchester, United Kingdom; Our Future Health, Manchester, United Kingdom; Our Future Health, Manchester, United Kingdom; Our Future Health, Manchester, United Kingdom; Institute of Psychiatry, Psychology and Neuroscience, King’s College London, London, United Kingdom; Our Future Health, Manchester, United Kingdom; Our Future Health, Manchester, United Kingdom; Our Future Health, Manchester, United Kingdom; Our Future Health, Manchester, United Kingdom; Our Future Health, Manchester, United Kingdom; Our Future Health, Manchester, United Kingdom; Our Future Health, Manchester, United Kingdom; Our Future Health, Manchester, United Kingdom; Our Future Health, Manchester, United Kingdom; Our Future Health, Manchester, United Kingdom; Nuffield Department of Population Health, University of Oxford, Oxford, United Kingdom; Department of Epidemiology, Harvard T.H. Chan School of Public Health, Boston, MA, United States; Our Future Health, Manchester, United Kingdom; Our Future Health, Manchester, United Kingdom; Our Future Health, Manchester, United Kingdom; Our Future Health, Manchester, United Kingdom; Our Future Health, Manchester, United Kingdom; Our Future Health, Manchester, United Kingdom; Our Future Health, Manchester, United Kingdom; Our Future Health, Manchester, United Kingdom; Our Future Health, Manchester, United Kingdom; Our Future Health, Manchester, United Kingdom; EveryOne Medicines, Inc., London, United Kingdom; Institute of Psychiatry, Psychology and Neuroscience, King’s College London, London, United Kingdom; OLS/NIHR Mental Health Mission, London, United Kingdom; Department of Behavioural Science and Health, University College London, London, United Kingdom; Our Future Health, Manchester, United Kingdom; Our Future Health, Manchester, United Kingdom; Our Future Health, Manchester, United Kingdom; Nuffield Department of Population Health, University of Oxford, Oxford, United Kingdom; Our Future Health, Manchester, United Kingdom; Our Future Health, Manchester, United Kingdom; MRC Epidemiology Unit, University of Cambridge, Cambridge, United Kingdom

**Keywords:** cohort study, biobank, research volunteers selection, epidemiology, translational research, genetic research, pharmacoepidemiology, electronic health records, surveys and questionnaires, population health

Key Features

- Our Future Health is the UK’s largest health research programme with an ambition to recruit 5 million UK-resident adult participants into a prospective cohort study.
- Our mission is to help everyone live longer, healthier lives through the discovery and testing of more effective approaches to the prevention, earlier detection, and treatment of diseases.
- Recruitment opened in October 2022. By June 2025, 2.4 million participants had consented, 1.8 million had completed the baseline questionnaire, 1.3 million had been linked to electronic health records, and 1.4 million had completed an in-person appointment to donate health metrics and blood.
- Genotyping is conducted using a 700 000-variant custom array that is optimized for multi-ethnic imputation and has up-to-date disease-, phenotype-, blood type-, and pharmacogenomic-associated variants.
- The cohort includes >600 000 people aged 18–40 years, facilitating research into diseases that disproportionately affect younger people, including obesity, mental health disorders, and addiction, and 900 000 people aged >60 years, enabling research into vascular dementia, Alzheimer’s, and ageing.
- Our Future Health will catalyse translational research by facilitating access to stored biosamples and by enabling re-contact studies and trials with participants selectively invited based on demographics, phenotypes, and disease risks.
- To conduct research using the Our Future Health resource, please visit https://research.ourfuturehealth.org.uk/.

## Why was the cohort set up?

**Figure 1.**
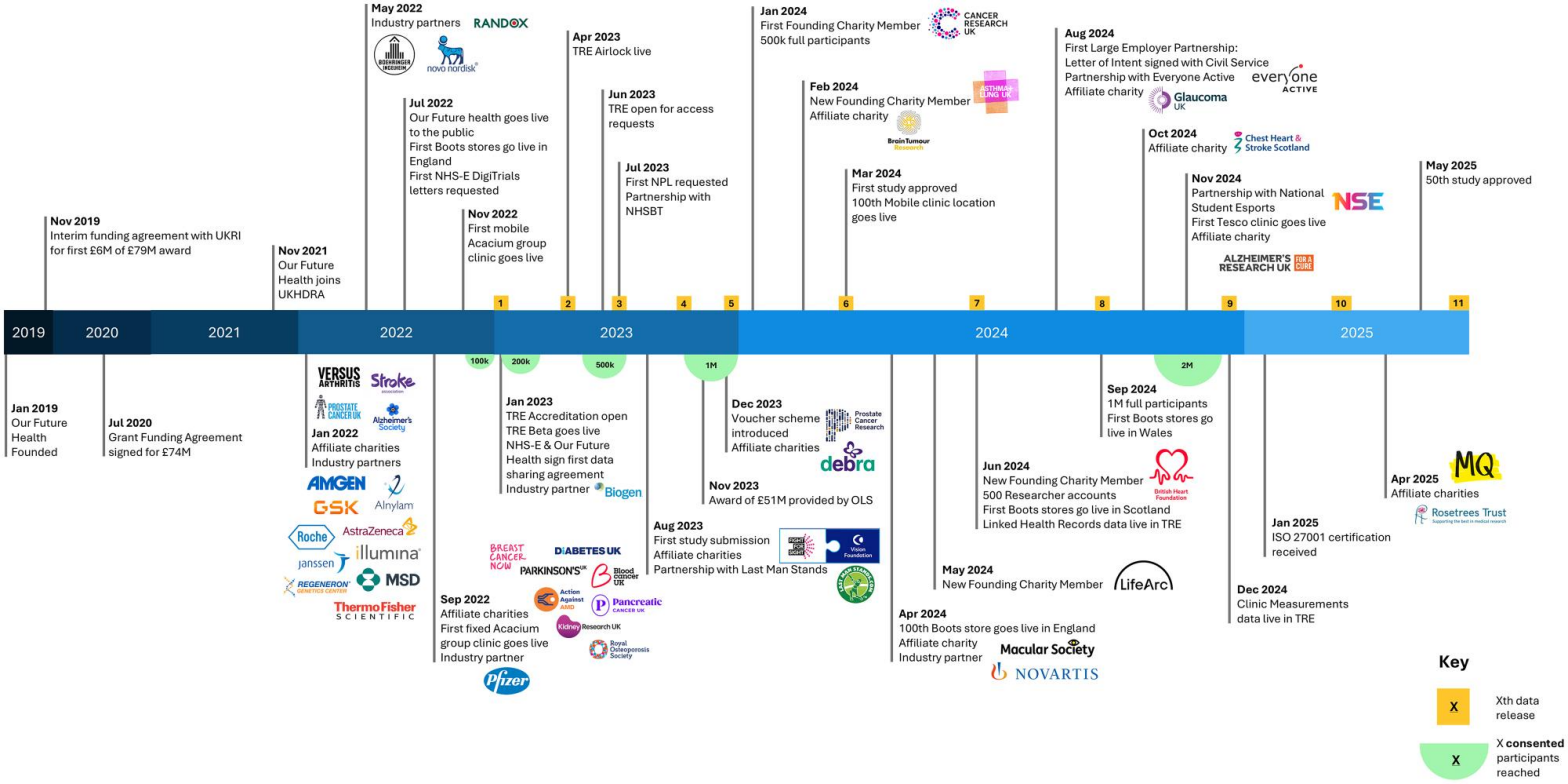
Timeline of Our Future Health showing participant milestones, major partnerships, and data releases. Green circles = number of consented participants. Yellow blocks = data releases. NHSBT, National Health Service Blood and Transplant; NHS-E, National Health Service—England; NPL, non-personalized letter; OLS, Office for Life Sciences; UKHDRA, UK Health Data Research Alliance; UKRI, UK Research and Innovation).

Our Future Health is a new UK-wide prospective cohort supported by the UK Government, industry, and charity sectors (Figure 1). Our objective is to recruit 5 million adult residents of the UK to facilitate aetiologic and translational research. This research will enable everyone to live longer, healthier lives by accelerating the discovery and testing of better ways to prevent, detect, and treat diseases.

Our Future Health also aims to recruit a diverse cohort that reflects the UK population. Ethically, it is important that we make participation in Our Future Health as equitable and accessible as possible. Scientifically, it is important that the participants are sufficiently diverse to facilitate a range of discovery and translational research, so that the benefits of research can be realized by all segments of the UK population.

Combined with a proportional representation of participants across the nations, Our Future Health will provide a truly diverse cohort that will be amenable to a variety of studies that have not been possible in a UK prospective cohort before, providing sufficient statistical precision to study:

- common and rare phenotypes and diseases;
- subpopulations based on ethnicity, index of multiple deprivation, geography, genotypes, precursor conditions, and disease risks;
- statistical interactions such as how genotypes modify therapeutic interventions;
- pre-diagnostic/pre-interventional bloods for studies of subpopulations with specific diseases/phenotypes;
- selected populations invited and consented into ancillary studies and trials.

Combining this large-scale diversity with appropriate sets of statistical weights will enhance the ability to make health-related inferences for local, country-specific, and national populations.

Our Future Health will also facilitate access to smaller sets of biospecimens to enable the discovery and validation of disease-specific biomarkers to help diagnose disease earlier as well as predict how the disease may progress. Participant consent for re-contact based on health-related information will also enable selective invitation to additional studies and trials, acting as a catalyst for clinical translation. The scale of Our Future Health will bring many rarer diseases into play for aetiological and translational research.

Our Future Health was set up with funding from the Industrial Strategy Challenge Fund, delivered by Innovate UK, and is an ambitious collaboration between the public sector, life sciences companies, and leading UK health charities. A full listing of our funders can be seen on our website (https://ourfuturehealth.org.uk/).

## Who is in the cohort?

Our Future Health is a non-probability, volunteer-based prospective cohort. The sample frame is the total UK-resident adult population. The recruitment strategy will provide large numbers of participants from the most deprived communities as well as from the primary ethnic minority groups resident in the UK: Indian, Pakistani, Bangladeshi, Chinese, Black African, Black Caribbean, Arab, and Mixed—populations that have been historically under-represented in health research. The recruitment strategy will also facilitate studies of deprivation, rural–urban indices, population density, and many other population and geographical variables that are associated with health.

Potential participants do not need to receive a formal invitation to join but, to create awareness and engagement, Our Future Health writes to invite all individuals or households, typically within a 5-mile radius, prior to opening new in-person appointment centres. We use a combination of personalized letters sent via National Health Service (NHS) DigiTrials (https://digital.nhs.uk/services/nhs-digitrials) and non-personalized letters, which include an invitation to join the programme and a QR code and web address to find out more. The number of letters and frequency depend on the number of available appointments. Awareness and engagement are supported by advertising in local and national media, and outreach to local community stakeholders.

Whether via the details in an invitation letter or any other route, any interested UK-resident adult can go to the Our Future Health website, read the consent and participant information sheet, decide whether they wish to register, and then consent to becoming a participant. After consent, participants can complete the baseline questionnaire and book an in-person appointment to provide health metrics and a blood sample.

Between 2020 and 2021, 120 members of the public were involved in the co-design of the protocol, participant information sheet, consent form, and other public-facing materials via focus groups, co-design meetings, and interviews. Our Future Health has continued to use learning from the public and participants to design the programme via qualitative, user research or co-design methods with >1000 individuals (as of January 2025) and with >500 000 participants completing a post-consent survey of their experience of the programme.

**Figure 2.**
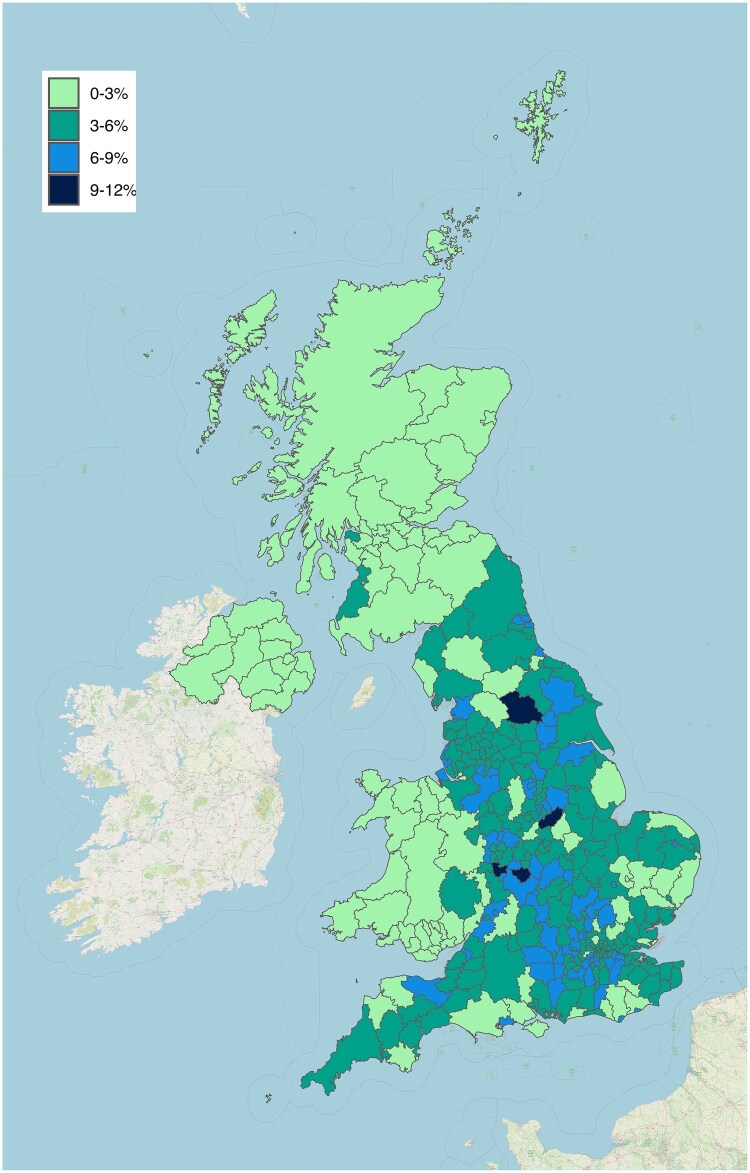
Percentages of consented adult populations by 2019 local authority districts (data as of June 2025). Mapped using OpenStreetMap [8].

Our Future Health initiated recruitment in October 2022. By June 2025, 2.4 million participants had consented (Figure 2), 1.8 million had completed the baseline questionnaire, 1.3 million had been linked to electronic health records, and 1.4 million had completed an in-person appointment to donate health metrics and blood. We estimate our response rate to be 4.5% based on the proportion who consent from the individuals/households we write to. We have optimized the response rate to the initial invitation, online sign-up journey, and in-person appointment attendance rates by using behavioural science and user testing methods with the public and our participants.

Table 1 shows the demographics of the current Our Future Health population compared with censuses conducted in 2021 (England and Wales, and Northern Ireland) and 2022 (Scotland). Recruitment invitation and in-person appointments started in England in October 2022 and in Wales and Scotland in early 2024. The distributions for age, sex, and ethnicity show the size and diversity of the cohort, which will provide broad research utility. In addition, the distributions of these demographics are reflective of the UK census distributions [1–3]. As we continue recruitment expansion in Scotland and Wales—and subsequently in Northern Ireland—the numbers and proportions of participants from these nations will become more aligned with the proportions from the most recent censuses.

**Table 1.** Recruitment numbers by age, sex, ethnicity, deprivation, and nation for different recruitment stages, from consented to full participant (completed questionnaire and usable blood sample)

Characteristic	Recruitment numbers (3 June 2025)								**UK Census 2021/2022** ^d^
	*Consented participants*		*Completed questionnaire*		*Has usable sample*		*Full participant*		
	*N*	%	*N*	%	*N*	%	*N*	%	%
**Total**	2 461 601	100.0	1 859 888	100.0	1 490 018	100.0	1 418 749	100.0	
Age group (years)^a^									
18–29	246 385	10.0	164 404	8.8	113 604	7.6	106 352	7.5	18.7
30–39	411 695	16.7	272 543	14.7	204 627	13.7	190 695	13.4	17.1
40–49	430 370	17.5	293 262	15.8	232 807	15.6	215 828	15.2	15.9
50–59	507 595	20.6	390 512	21.0	315 957	21.2	300 795	21.2	17.3
60–69	512 390	20.8	432 567	23.3	363 050	24.4	352 165	24.8	13.7
70–79	295 451	12.0	258 746	13.9	219 921	14.8	214 514	15.1	11.0
≥80	57 715	2.3	47 854	2.6	40 052	2.7	38 400	2.7	6.3
Sex at birth^b^									
Female	1 147 547	46.6	1 065 345	57.3	800 008	53.7	787 351	55.5	51.7
Male	851 324	34.6	793 428	42.7	644 802	43.3	630 640	44.5	48.3
Other	1 487	0.1	1 115	0.1	806	0.1	758	0.1	–
Unknown	461 243	18.7	0	0.0	44 402	3.0	0	0.0	–
Ethnicity^b^									
Asian	113 315	4.6	100 195	5.4	78 241	5.3	74 772	5.3	7.9
Black	33 130	1.3	28 189	1.5	21 946	1.5	20 667	1.5	3.3
White	1 772 179	72.0	1 673 311	90.0	1 297 911	87.1	1 280 558	90.3	85.1
Mixed	35 619	1.4	32 224	1.7	24 229	1.6	23 468	1.7	1.8
Other	30 600	1.2	25 969	1.4	20 611	1.4	19 284	1.4	1.9
Unknown	476 758	19.4	0	0.0	47 080	3.2	0	0.0	–
IMD^c^									
1—most deprived	321 738	13.1	221 139	11.9	165 932	11.1	154 124	10.9	20.0
2	402 687	16.4	292 611	15.7	231 442	15.5	217 814	15.4	20.0
3	458 793	18.6	347 752	18.7	278 134	18.7	264 948	18.7	20.0
4	551 194	22.4	428 467	23.0	347 167	23.3	332 413	23.4	20.0
5—least deprived	652 738	26.5	518 544	27.9	429 405	28.8	413 609	29.2	20.0
Unknown	74 451	3.0	51 375	2.8	37 938	2.5	35 841	2.5	–
Nation									
England	2 394 827	97.3	1 812 113	97.4	1 459 544	98.0	1 389 043	97.9	84.3
Scotland	40 933	1.7	30 434	1.6	20 693	1.4	20 211	1.4	8.2
Wales	19 247	0.8	14 196	0.8	9 361	0.6	9 138	0.6	4.6
Northern Ireland	1 021	0.0	562	0.0	50	0.0	48	0.0	2.8
Other/unknown	5 573	0.2	2 583	0.1	370	0.0	309	0.0	–

Participants can answer the questionnaire and attend an in-person appointment in either order. We continue to follow up individuals who have completed the whole journey to ensure that full data are ascertained in as short a time window as possible.

a Age group calculated for recruitment numbers based on year of birth and for data release based on month and year of birth.

b For sex and ethnicity, we only have data if participant started the questionnaire.

c Adjusted composite Index of Deprivation score based on Parsons, Alex (2021), UK 2020 Composite Index of Multiple Deprivation, https://github.com/mysociety/composite_uk_imd.

d Combined census data for England, Scotland, Wales, and Northern Ireland from 2021 and 2022 for adults (>18 years).

IMD, Index of Multiple Deprivation; TRE, Trusted Research Environment. A dash indicates that data are not available from the census in this category.

## How often have they been followed up?

Our Future Health participants consent to health-related data linkages, broad analysis of their donated and biobanked blood samples, to be re-contacted to be invited to complete additional surveys or provide additional biospecimens, to take part in ancillary research studies, and to receive feedback about their risks of diseases in the future. These design features provide a broad range of ways to follow-up participants and maximize translational research opportunities within the programme.

**Figure 3.**
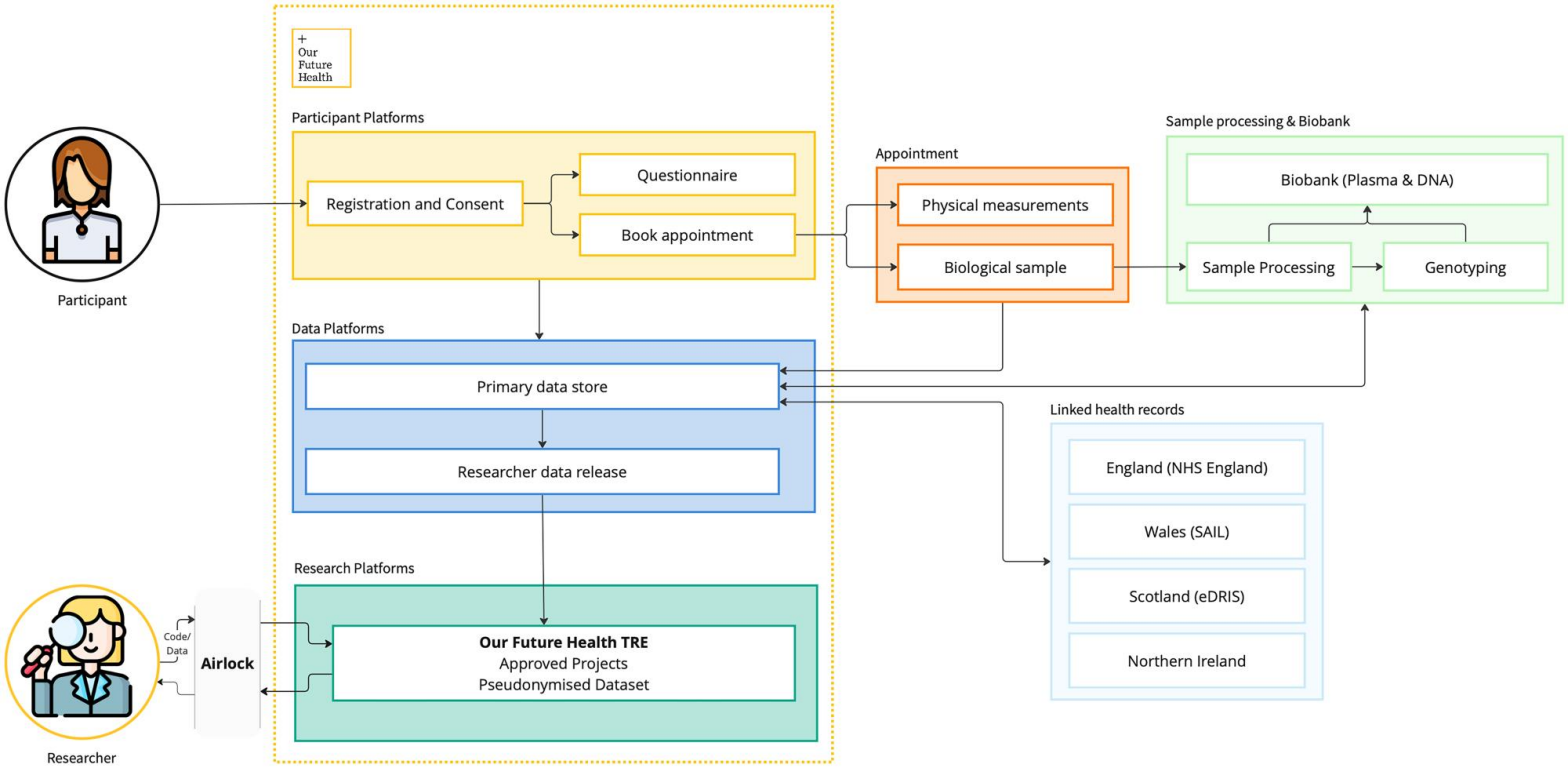
Schematic overview of current data and biological samples. An airlock is a secure method of transferring files into and out of the platform space. eDRIS, Electronic Data Research and Innovation Service.

Data linkages to NHS England secondary care, cancer registry, and death registry databases have been established with a current matching success of 96.1% (despite also including participants with a study baseline residence in Scotland, Wales, or Northern Ireland, which may be less likely to have an NHS England record). NHS England secondary care includes admitted patient care, outpatient, accident and emergency, critical care, and emergency care datasets, while NHS England cancer data include cancer registry, treatment, and patient pathways. Our Future Health is in the process of establishing similar data linkages with NHS Scotland and with the Wales Secure Anonymised Information Linkage Databank (SAIL) Databank (the latter of which includes primary care data) with applications currently under review. We will apply for primary care data in England and Scotland when these become available. For Northern Ireland, we are currently building our data-linkage strategy for these four initial high-value datasets (primary and secondary care, cancer, and death registrations). Linkages will be updated on a regular basis to provide prospectively accrued event and outcome data (Figure 3).

Participant blood samples are already used for genotyping on a custom 700 000-variant array. Biobanked plasma, buffy coat, and deoxyribonucleic acid (DNA) will enable Our Future Health to conduct additional omics assays (e.g. proteomics, whole-genome sequencing, methylation) on the whole cohort or sub-cohorts. Our Future Health will also provide access to biobanked samples to external researchers for approved studies, facilitating large, omics-based analyses as well as smaller-scale, disease-focused studies.

Our Future Health will, in future, send further questionnaires to all participants to ascertain additional exposures (e.g. cognitive function, diet, pain, COVID-19, geolocation data), to assess reporting errors, and for the repeat ascertainment of time-varying exposures. In addition, selective invitation based on health-related data that we hold is permitted. This will enable Our Future Health, as well as external research study investigators, to select participants for invitations to additional studies, providing a huge leap in the efficiencies of matching specific participants to tailored studies, maximizing benefits for all.

## What has been measured?

Our data documentation on what has been measured is available on our dedicated researcher website (https://research.ourfuturehealth.org.uk/data-and-cohort/).

After consent, participants are asked to complete a five-module baseline questionnaire that takes ∼35 minutes and captures information on personal demographics and household, work and education, lifestyle exposures, family health history, and personal health history (see the questionnaire in our Study Protocol Appendix at https://research.ourfuturehealth.org.uk/ or on our GitBook site at https://ourfuturehealth.gitbook.io/our-future-health). Participants are also invited to book a 15-minute in-person appointment, at a time and location of their preference from among our active centres. At the appointment, they provide health information [height, weight, waist circumference, blood pressure, heart rate, point-of-care cholesterol test (for the initial 1.2 million participants from 2022–2024)], and a blood sample (2 × 6-ml EDTA [ethylenediaminetetraacetic acid] vials).

Anthropometric and cardiac measurements are available to access now. Height was measured by using the Marsden HM-250P stadiometer, weight by using SECA 875 scales, and cardiac measurements by using either an Omron 3 BP monitor or an Omron HBP 1320.

Blood samples are couriered overnight at ambient temperature before being centrifuged (2500 rpm for 10 minutes at 4°C) and aliquoted into 6 × 850-µl vials of plasma and 1 × 500-µl vial of buffy coat, with the other buffy coat used for DNA extraction. Overnight ambient logistics was required due to the rapid and highly dispersed recruitment requirements of the programme. This strategy was also supported by prior evidence showing that only a small proportion of metabolites were markedly affected by this protocol compared with immediate processing [4–6]. Biosamples are stored in fully automated −80°C freezers. DNA is extracted by using magnetic bead technologies (Revvity Chemagen, Chemagic-360 or ThermoFisher Scientific, KingFisher). DNA quality and quantity are assessed by using spectral absorbance.

Participant DNA samples are genotyped by using a custom 700 000-variant Illumina Infinium Excalibur beadchip array assay. This was designed by Our Future Health in collaboration with Illumina, drawing on expertise from across the academic, life sciences, and charity sectors. The design also included considerations of health equity by using lower *P*-value thresholds for disease-associated variants from studies with predominantly non-European populations and optimizing the design for UK-population multi-ethnic imputation. The design includes up-to-date disease-, phenotype-, blood type-, and pharmacogenomic-associated variants. The genotype array variant file—in plain-text csv format providing chrom-pos-ref-alt details from GRCh38—is available on our researcher website. Genetic data for >650 000 participants are already available for researchers in our Trusted Research Environment (TRE).

For the point-of-care cholesterol test, the Mission^®^ Cholesterol Lipid Pro device was used (ACON Laboratories, Inc., San Diego, USA). The device measures total cholesterol, high-density lipoprotein, and triglycerides, and estimates low-density lipoprotein. At the time of selection, this device was one of only four that met the National Cholesterol Education Program required standard of evidence for bias and precision [7] and we independently verified its high sensitivities and specificities compared with a laboratory gold standard (Roche Cobas 702) by using 264 non-fasted blood samples. The Mission device has been used during 2022–2024 for the first 1.2 million in-person appointments and these data will be made available for researchers in a forthcoming data release.

Our Future Health will work with collaborators to expand omics datasets available in the resource and will also open for sample access requests for disease-specific biomarker research proposals. These data will further enrich the resource and further broaden the research potential of the programme.

## What has it found?

Our Future Health is a diverse cohort of UK adults. Of the ∼1.86 million who to date have consented to join the programme and submitted a questionnaire (Table 1), 23.5% are <40 years of age and 39.8% are >60 years of age (versus 35.8% and 31%, respectively, on the combined 2021/2022 UK Censuses). In addition, 57.3% are female (versus 51.7% on the census) and 10% are of non-White ethnicity (versus 14.9% on the census).

The questionnaire provides a contemporary picture of participants’ health and lifestyles. This includes recent trends such as vaping (use of e-cigarettes), with 46% of participants aged 18–29 years reporting ever vaping compared with just 4.5% of participants aged ≥60 years (Table 2). Highlighting the burden of ill health and potential for better detection, prevention, and treatment of disease, a large proportion of participants (69.8%) reported having had at least one of a broad panel of current or past health conditions (Supplementary Tables S1 and S2). As expected, the proportion of participants reporting at least one condition was higher among older people (79.9% of those aged ≥60 years) but, even among those aged 18–29 years, the majority (59.0%) reported at least one condition. Multiple long-term conditions were also reported by 39.4% of participants.

**Table 2.** Characteristics of the Our Future Health cohort from the 11th data release in June 2025.

Characteristic	Total (*N* = 1 781 891)		Female (*N* = 1 020 110)		Male (*N* = 760 735)		Aged 18–29 years (*N* = 167 728)		Aged 30–59 years (*N* = 925 642)		Aged ≥60 years (*N* = 688 521)	
	Mean	SD	Mean	SD	Mean	SD	Mean	SD	Mean	SD	Mean	SD
Self-reported measurements												
Weight (kg)	77.4	18.0	71.3	16.8	85.3	16.2	74.0	18.6	78.9	18.6	76.3	16.7
Height (cm)	169.9	9.9	164.0	6.9	177.8	7.3	169.9	9.8	170.3	9.9	169.5	9.9
BMI (kg/m^2^)	26.7	5.6	26.5	6.1	27.0	4.9	25.5	6.0	27.1	5.8	26.5	5.1

	***N***	**%**	***N***	**%**	***N***	**%**	***N***	**%**	***N***	**%**	***N***	**%**

**Socioeconomic characteristics**												
*Annual household income*												
<£18 000	169 606	9.5	110 297	10.8	59 200	7.8	15 277	9.1	64 842	7.0	89 487	13.0
£18 000–£30 999	271 545	15.2	160 852	15.8	110 578	14.5	21 426	12.8	98 747	10.7	151 372	22.0
£31 000–£51 999	366 048	20.5	204 630	20.1	161 295	21.2	31 101	18.5	173 100	18.7	161 847	23.5
£52 000–£100 000	462 024	25.9	250 621	24.6	211 300	27.8	46 124	27.5	301 380	32.6	114 520	16.6
>£100 000	223 484	12.5	112 383	11.0	111 053	14.6	16 564	9.9	172 571	18.6	34 349	5.0
Do not know	79 131	4.4	54 998	5.4	24 054	3.2	29 607	17.7	28 804	3.1	20 720	3.0
Prefer not to answer	210 053	11.8	126 329	12.4	83 255	10.9	7 629	4.5	86 198	9.3	116 226	16.9
*Qualifications (highest level)*												
College or university degree	920 232	51.6	534 484	52.4	385 354	50.7	101 220	60.3	536 744	58.0	282 268	41.0
A-levels/AS-levels/BTEC or equivalent	293 223	16.5	166 867	16.4	126 226	16.6	45 350	27.0	145 367	15.7	102 506	14.9
O-levels, GCSEs, or equivalent	276 641	15.5	164 761	16.2	111 801	14.7	12 075	7.2	127 401	13.8	137 165	19.9
CSEs or equivalent	75 361	4.2	41 035	4.0	34 309	4.5	768	0.5	31 495	3.4	43 098	6.3
NVQ, HND, or HNC or equivalent	63 390	3.6	29 422	2.9	33 938	4.5	2415	1.4	30 651	3.3	30 324	4.4
Other professional qualifications, e.g. nursing, teaching	40 664	2.3	24 723	2.4	15 925	2.1	950	0.6	14 301	1.5	25 413	3.7
None of the above	85 760	4.8	44 576	4.4	41 127	5.4	2754	1.6	25 037	2.7	57 969	8.4
Prefer not to answer/missing	26 620	1.5	14 242	1.4	12 055	1.6	2196	1.3	14 646	1.6	9 778	1.4
**Lifestyle characteristics**												
*Exercise*												
Walk for ≥10 minutes ≥4 times per week	1 426 167	80.0	817 759	80.2	607 825	79.9	136 604	81.4	730 850	79.0	558 713	81.1
Moderate physical activity ≥4 times per week	868 770	48.8	483 099	47.4	385 313	50.7	72 670	43.3	430 529	46.5	365 571	53.1
Vigorous physical activity ≥4 times per week	391 698	22.0	197 624	19.4	193 897	25.5	37 894	22.6	211 269	22.8	142 535	20.7
*Cigarette-smoking status*												
Current smoker	122 519	6.9	66 704	6.5	55 723	7.3	18 453	11.0	80 003	8.6	24 063	3.5
Former smoker	520 000	29.2	281 615	27.6	238 176	31.3	18 289	10.9	254 860	27.5	246 851	35.9
Never smoker	1 109 018	62.2	655 989	64.3	452 515	59.5	126 607	75.5	573 330	61.9	409 081	59.4
Prefer not to answer/missing	30 354	1.7	15 802	1.5	14 321	1.9	4379	2.6	17 449	1.9	8526	1.2
*Vaping status*												
Ever used	284 633	16.0	162 937	16.0	121 553	16.0	77 223	46.0	176 462	19.1	30 948	4.5
Never used	1 484 122	83.3	850 444	83.4	632 986	83.2	88 560	52.8	741 117	80.1	654 445	95.1
Prefer not to answer	13 136	0.7	6729	0.7	6196	0.8	1945	1.2	8063	0.9	3128	0.5
**Health characteristics**												
*Current health status*												
Poor	98 448	5.5	59 416	5.8	38 931	5.1	10 744	6.4	58 009	6.3	29 695	4.3
Fair	429 811	24.1	241 996	23.7	187 567	24.7	42 164	25.1	226 035	24.4	161 612	23.5
Good	985 211	55.3	566 420	55.5	418 384	55.0	87 301	52.0	500 812	54.1	397 098	57.7
Excellent	259 126	14.5	147 050	14.4	111 987	14.7	26 005	15.5	135 414	14.6	97 707	14.2
Do not know/prefer not to answer	9294	0.5	5227	0.5	3866	0.5	1513	0.9	5372	0.6	2409	0.3

BMI, body mass index; BTEC, Business and Technology Education Council; CSEs, Certificate of Secondary Education; GCSEs, General Certificate of Secondary Education; NVQ, National Vocational Qualification; HND, Higher National Diploma; HNC, Higher National Certificate.

The first approved research studies became active in the Our Future Health TRE in early 2024. At the time of writing, there were 43 studies active in the TRE, summaries of which can be found on the HDR UK Health Data Research Gateway (https://healthdatagateway.org/en/data-custodian/86). Further extensive documentation on the study design, recruited cohort, and data releases, including the latest descriptive summaries of data, data dictionaries, and other details of the questionnaire, linked health records, and genotype array data, can be found on the Our Future Health researcher website (https://research.ourfuturehealth.org.uk/) and our GitBook site (https://ourfuturehealth.gitbook.io/our-future-health).

## What are the main strengths and weaknesses?

One of the major strengths of Our Future Health is its large size, which provides a singular, prospective cohort with sufficient statistical precision to study:

- common and rare phenotypes and diseases;
- subpopulations based on ethnicity, index of multiple deprivation, geography, genotypes, precursor conditions, and disease risks;
- statistical interactions such as how genotypes modify therapeutic interventions;
- pre-diagnostic/pre-interventional bloods for studies of subpopulations with specific diseases/phenotypes.

In addition, the Our Future Health research programme has been designed to facilitate translational research with the forthcoming ability for study proposals to include access to baseline blood samples and for investigator-led re-contact/additional studies to selectively invite participants based on demographics, phenotypes, genotypes, or disease risks for interventional research, clinical trials, and patient-centred research.

One of the weaknesses to be aware of is selection bias in the form of participant self-selection. This is a problem in all non-probability, volunteer-based recruitment programmes. It is an inherent nature of this design, which is necessitated by the scale and efficiencies required by the programme. However, Our Future Health has successfully recruited a cohort that is widely reflective of the diversity of the UK population. This will help minimize selection biases and maximize the participation of groups in the UK who have been historically under-represented in health research. We will further mitigate the risk of this form of selection bias by providing sampling weights and statistical code to improve the accuracy of statistical inferences to UK populations in analyses of data from Our Future Health.

## Can I get hold of the data? Where can I find out more?

The Our Future Health resource can be accessed by researchers across the globe for the purpose of health research that is for the public good. Researchers must meet the requirements set out in our transparent and equitable access process that ensures the security of the data and trust placed in us by our participants. Interested researchers create an account and submit an application to become registered with Our Future Health. After approval, registered researchers can then submit a study application that will be reviewed by our Access Board. Following approval and access fee payment, access to the data required for the study is then enabled in a TRE.

Further information about the access process, including details of the steps involved, latest available data, detailed documentation, and how to contact support, can be found through our dedicated researcher website at https://research.ourfuturehealth.org.uk/.

## Ethics approval

The research programme received favourable opinion from the East of England—Cambridge East Research Ethics Committee (REC reference: 21/EE/0016, https://www.hra.nhs.uk/planning-and-improving-research/application-summaries/research-summaries/our-future-health) on 29 March 2021 for a period of 5 years.

## Supplementary Material

dyaf171_Supplementary_Data

## Data Availability

See the above section “Can I get hold of the data? Where can I find out more?” for more details.
