# Supplementary material for: Cohort Profile: Our Future Health
Source: Int J Epidemiol. 2025 Oct 15;54(6):dyaf171. doi: 10.1093/ije/dyaf171 (PMC12527338; doi:10.1093/ije/dyaf171)
Supplement: dyaf171_Supplementary_Data [file dyaf171_supplementary_data.docx]

**Supplementary Material**

Table S1: Self-reported medical conditions

| **Categorised Medical Diagnoses** | **Total** | | **Female** | | **Male** | | **Aged 18-29** | | **Aged 30-59** | | **Aged 60+** | |
| --- | --- | --- | --- | --- | --- | --- | --- | --- | --- | --- | --- | --- |
|  | **(N = 1,729,465)** | | **(N = 990,568)** | | **(N = 737,881)** | | **(N = 162,601)** | | **(N = 898,100)** | | **(N = 668,764)** | |
|  | **n** | **%** | **n** | **%** | **n** | **%** | **n** | **%** | **n** | **%** | **n** | **%** |
| Have you ever been diagnosed with any of the following by a doctor or other health professional? | | | | | | | | | | | | |
| Autoimmune disorder | 91,548 | 5.3 | 68,254 | 6.9 | 23,254 | 3.2 | 5,737 | 3.5 | 49,914 | 5.6 | 35,897 | 5.4 |
| Blood disorders (Anaemia) | 125,796 | 7.3 | 105,729 | 10.7 | 20,014 | 2.7 | 12,541 | 7.7 | 65,361 | 7.3 | 47,894 | 7.2 |
| Cancer | 133,276 | 7.7 | 75,119 | 7.6 | 58,118 | 7.9 | 727 | 0.5 | 35,283 | 3.9 | 97,266 | 14.5 |
| Digestive system or liver problems | 209,401 | 12.1 | 129,822 | 13.1 | 79,495 | 10.8 | 13,758 | 8.5 | 101,579 | 11.3 | 94,064 | 14.1 |
| Endocrine, nutritional and metabolic disorders (e.g. diabetes, thyroid disorder, vitamin deficiencies) | 184,745 | 10.7 | 136,059 | 13.7 | 48,600 | 6.6 | 11,754 | 7.2 | 90,180 | 10.0 | 82,811 | 12.4 |
| Eye or visual problems | 312,364 | 18.1 | 171,788 | 17.3 | 140,426 | 19.0 | 17,715 | 10.9 | 119,297 | 13.3 | 175,352 | 26.2 |
| Fractures, breaks, or joint problems | 397,007 | 23.0 | 225,617 | 22.8 | 171,237 | 23.2 | 20,977 | 12.9 | 172,095 | 19.2 | 203,935 | 30.5 |
| Heart or circulatory disease (e.g. high blood pressure or stroke) | 279,144 | 16.1 | 129,981 | 13.1 | 149,051 | 20.2 | 4,506 | 2.8 | 89,455 | 10.0 | 185,183 | 27.7 |
| Kidney or urinary system disorders | 107,732 | 6.2 | 57,073 | 5.8 | 50,609 | 6.9 | 4,171 | 2.6 | 38,709 | 4.3 | 64,852 | 9.7 |
| Lung or respiratory problems | 190,248 | 11.0 | 111,088 | 11.2 | 79,074 | 10.7 | 15,910 | 9.8 | 90,328 | 10.1 | 84,010 | 12.6 |
| Mental health conditions (e.g. depression, bipolar disorder) | 343,095 | 19.8 | 239,536 | 24.2 | 103,348 | 14.0 | 49,260 | 30.3 | 206,921 | 23.0 | 86,914 | 13.0 |
| Neurodevelopmental conditions (e.g. Autism spectrum disorder, ADHD) | 37,746 | 2.2 | 23,891 | 2.4 | 13,775 | 1.9 | 13,499 | 8.3 | 21,871 | 2.4 | 2,376 | 0.4 |
| Neurological disorders (things that affect that brain or nervous system. E.g., Epilepsy) | 43,141 | 2.5 | 27,456 | 2.8 | 15,654 | 2.1 | 4,369 | 2.7 | 22,966 | 2.6 | 15,806 | 2.4 |
| Reproductive system problems | 104,394 | 6.0 | 91,198 | 9.2 | 13,167 | 1.8 | 9,972 | 6.1 | 62,044 | 6.9 | 32,378 | 4.8 |

*These counts—which are categorised from more granular self-reported medical diagnoses—are from version 2 of the questionnaire; the first 52,426 participants answered version 1 of the questionnaire which had a less comprehensive medical condition section.*

Table S2: Self-reported medications.

| **Categorised Medication** | **Total** | | **Female** | | **Male** | | **Aged 18-29** | | **Aged 30-59** | | **Aged 60+** | |
| --- | --- | --- | --- | --- | --- | --- | --- | --- | --- | --- | --- | --- |
|  | **(N = 1,729,465)** | | **(N = 990,568)** | | **(N = 737,881)** | | **(N = 162,601)** | | **(N = 898,100)** | | **(N = 668,764)** | |
|  | **n** | **%** | **n** | **%** | **n** | **%** | **n** | **%** | **n** | **%** | **n** | **%** |
| Do you regularly take medications for any of the following reasons? | | | | | | | | | | | | |
| Autoimmune disorder | 45,955 | 2.7 | 33,243 | 3.4 | 12,692 | 1.7 | 2,590 | 1.6 | 25,112 | 2.8 | 18,253 | 2.7 |
| Bone health | 70,562 | 4.1 | 57,354 | 5.8 | 13,184 | 1.8 | 880 | 0.5 | 17,117 | 1.9 | 52,565 | 7.9 |
| Cancer | 19,142 | 1.1 | 13,337 | 1.4 | 5,798 | 0.8 | 119 | 0.1 | 6,693 | 0.8 | 12,330 | 1.8 |
| Diabetic health | 57,100 | 3.3 | 22,542 | 2.3 | 34,518 | 4.7 | 1,035 | 0.6 | 20,551 | 2.3 | 35,514 | 5.3 |
| Digestive problems (including acid reflux and liver problems) | 204,026 | 11.8 | 118,851 | 12.0 | 85,088 | 11.5 | 7,738 | 4.8 | 79,551 | 8.9 | 116,737 | 17.5 |
| Endocrine disorder (e.g. under or over-active thyroid) | 84,485 | 4.9 | 70,246 | 7.1 | 14,205 | 1.9 | 2,504 | 1.5 | 38,574 | 4.3 | 43,407 | 6.5 |
| Heart or circulatory health (e.g. high blood pressure or stroke) | 302,569 | 17.5 | 131,862 | 13.3 | 170,589 | 23.1 | 1,988 | 1.2 | 80,039 | 8.9 | 220,542 | 33.0 |
| Lung or breathing problems | 126,293 | 7.3 | 76,769 | 7.8 | 49,453 | 6.7 | 9,740 | 6.0 | 60,343 | 6.7 | 56,210 | 8.4 |
| Mental health conditions or insomnia (e.g. depression, bipolar disorder) | 189,709 | 11.0 | 136,490 | 13.8 | 53,089 | 7.2 | 25,484 | 15.7 | 117,469 | 13.1 | 46,756 | 7.0 |
| Neurological disorders (e.g. Alzheimers, epilepsy, Parkinson's) | 22,354 | 1.3 | 13,627 | 1.4 | 8,719 | 1.2 | 2,256 | 1.4 | 11,424 | 1.3 | 8,674 | 1.3 |
| Pain relief | 270,157 | 15.6 | 186,558 | 18.8 | 83,464 | 11.3 | 19,091 | 11.7 | 133,449 | 14.9 | 117,617 | 17.6 |
| Reproductive or sexual health (including contraception, erectile dysfunction, menopause or hormone medication) | 136,485 | 7.9 | 110,362 | 11.1 | 26,045 | 3.5 | 20,244 | 12.5 | 84,606 | 9.4 | 31,635 | 4.7 |
| Supplements or nutritional health | 415,108 | 24.0 | 279,186 | 28.2 | 135,764 | 18.4 | 35,524 | 21.9 | 215,178 | 24.0 | 164,406 | 24.6 |

*These counts—which are categorised from more granular self-reported medications usage—are from version 2 of the questionnaire; the first 52,426 participants answered version 1 of the questionnaire which had a less comprehensive medication section.*
